# Pesticides: Carbofuran under Review

**DOI:** 10.1289/ehp.116-a425

**Published:** 2008-10

**Authors:** David Tenenbaum

In the 31 July 2008 *Federal Register*, the Environmental Protection Agency (EPA) published a proposal to revoke all food “tolerances” of the insecticide carbofuran—residue allowances that are allowed to appear on food—on both domestic and imported produce, based on excessive risks to children and wildlife. If approved, this reversal of a July 2006 decision to allow carbofuran tolerances on imported coffee, bananas, sugarcane, and rice would sidestep the need for all-out cancellation of the pesticide’s registration.

Carbofuran (trade name Furadan) is a restricted-use pesticide registered in the United States since 1969. The compound causes overstimulation of the nervous system by inhibiting acetyl-cholinesterase. Symptoms of overexposure in humans include headache, weakness, abdominal cramping, nausea, blurred vision, convulsion, tremor, and coma. Carbofuran is highly toxic to birds, fish, and bees.

The proposal to revoke tolerances was necessary to protect children, says agency spokesperson Dale Kemery, because “studies have consistently shown that juvenile rats are more sensitive to carbo-furan than adult rats.” Based on EPA risk assessments, children up to age 5 also “experience the highest levels of dietary risk because they tend to eat larger amounts of food per their body weight than do teenagers or adults and because their systems for detoxifying contaminants are still developing.”

That assessment was overly conservative, argues John Cummings, regulatory manager for North America at the Agricultural Products Group of FMC Corporation, the sole U.S. manufacturer of carbo-furan. Cummings says the EPA’s risk calculation includes the use of three safety factors, whereas FMC argues that the toxicologic data support a value of 100× for the combination of these factors but not the 400× asserted by the EPA. The three safety factors include a 10× interspecies factor used to extrapolate from animal data to humans, a 10× intraspecies factor used to account for possible variations between individuals, and a 4× uncertainty factor that is permissible under the Food Quality Protection Act.

The EPA is only reacting to the law, Kemery says: “We are required by section 408 of the Federal Food Drug and Cosmetic Act to take into account the potential for children to be more sensitive in establishing safe levels on food—for example, by including additional safety factors in our calculations.” The American Bird Conservancy, which has fought for several years to have carbo-furan banned, points out that as far back as 1980 EPA scientists had estimated that over a million birds were killed each year by the granular formulation of carbofuran, the registration for which was cancelled in 1994.

Beyond the tolerance revocation, the EPA embarked years ago on an effort to ban all U.S. sales of carbofuran by cancelling its registration altogether on the basis of dietary, occupational, and environmental considerations. This larger move has gotten support from several environmental groups. Alternatively, several U.S. grower groups and the U.S. Department of Agriculture have asked the EPA to consider alternatives to cancellation, claiming carbofuran is economically important to U.S. agriculture. About 1 million pounds are used annually on fruit and vegetable crops including corn, cotton, potatoes, sunflowers, and tobacco.

If, following the 29 September 2008 deadline for comments on the proposed action, the EPA issues a final tolerance revocation, FMC could request a hearing. If the revocation becomes law, it would become illegal to sell domestic or imported food bearing any residue of carbo-furan; enforcement would be the responsibility of the Food and Drug Administration.

